# A Systematic Study of the Effect of pH on the Initialization of Ca-deficient Hydroxyapatite to β-TCP Nanoparticles

**DOI:** 10.3390/ma12030354

**Published:** 2019-01-23

**Authors:** Feray Bakan

**Affiliations:** Sabanci University SUNUM Nanotechnology Research Center, Istanbul TR-34956, Turkey; feraybakan@sabanciuniv.edu; Tel.: +90-216-483-9000

**Keywords:** β-tricalcium phosphate, Ca-deficient hydroxyapatite, wet chemical precipitation, nanobiomaterials, nanocharacterization

## Abstract

The formation of β-tricalcium phosphate (β-TCP) nanoparticles via a wet precipitation technique was studied in a systematical way, taking reaction pH and sintering temperature parameters into account. A full transformation of Ca-deficient hydroxyapatite (CDHA) to β-TCP at 750 °C in under 3 h from Ca^++^ and PO_4_^3−^ precursor solutions prepared under a pH of 5.5 was observed. For pH values higher than 6.5, CDHA can only partially transform into β-TCP and only at temperatures higher than 750 °C confirmed using X-Ray diffraction and Raman spectroscopy. The morphologies of the particles were also examined by Transmission electron microscopy. The lower temperatures and the shorter sintering time allow for a fine needle-like morphology, but with a high crystallinity, likely eliminating the possibility of excessive grain growth that is otherwise expected to occur under high-temperature treatment with long process times. We show that sintering of nanostructured, high crystallinity β-TCP at relatively low temperatures is possible via adjustment of the precursor solution parameters. Such an outcome is important for the use of β-TCP with a fine morphology imitating that of the skeletal tissues, enhancing the osteointegration of a base, load-bearing alloy to the host tissue. MTT analysis was used to test the effect of the obtained β-TCP particles on the viability of MG-63 human osteoblast-like cells.

## 1. Introduction

Nano biomaterials have relatively recently found use in a variety of research and application fields, such as biomedicine [1], drug delivery [2], medical imaging [3], and cancer treatment [4,5]. Since the particle sizes of nanomaterials are small enough even to penetrate through cells, possible risks, such as cytotoxicity of these particles, should be considered carefully. Bio-nanomaterials include and are not limited to polymeric nanoparticles [6,7,8], metallic nanoparticles [9,10,11], and inorganic nanoparticles [12,13,14,15]. Among them, calcium phosphate (CaP) nanoparticles are preferred in many applications owing to their excellent biocompatibility, bioactivity, and chemical affinity towards biological molecules, such as nucleic acids, proteins, growth factors, etc. [16,17,18]. Hydroxyapatite (HA), β-Tricalcium phosphate (β-TCP), and biphasic calcium phosphates (BCP, mixtures of HA and β-TCP in a variety of ratios) are the most widely used CaP compounds [19]. HA is the major component of mammalian hard tissues and its synthetic forms are extensively utilized in a large spectrum of bio-applications [20]. Nevertheless, poor biodegradability of HA in the human body limits some of these applications [21,22]. To promote biological anchoring, a skeletal implant material is expected to be involved in complex healing processes, such as adhesion of the implant to the host tissue, new bone formation, and remodeling, etc. These biological processes are directly related to the chemical composition, surface charge, wettability, and roughness of the implanted material. When compared to HA, it has been posited that β-TCP exhibits better biodegradability, hence it can be absorbed better and replaced by newly generated hard tissues [21]. This property makes β-TCP a promising biomaterial among all other non-resorbable materials, and nano-sized β-TCP, in particular, has attracted great attention in many biomedical applications.

β-TCP can be prepared by means of the solid-state reaction of acidic CaPO_4_, e.g., dicalcium phosphate anhydrous, with a base, e.g., CaO [23,24,25,26], or wet chemical methods [19,27,28,29,30], both of which need to be followed by a heat treatment as β-TCP is a high-temperature phase. In wet-chemical processes, β-TCP cannot be directly precipitated, but can be transformed from a non-stoichiometric apatite, which has a molar ratio of Ca/P ranging from 1.33 to 1.65 [20,22] during heat treatment. Non-stoichiometric apatite with the formula, Ca_10−x_(HPO_4_)_x_(PO_4_)_6−x_(OH)_2−x_ (0 ≤ x ≤ 1), has a crystal structure similar to the stoichiometric HA. Heat treatment is required for non-stoichiometric apatite to be transformed into HA and β-TCP according to Equation (1):

(1) $${\mathrm{Ca}}_{10-x}{\left({\mathrm{HPO}}_{4}\right)}_{x}{\left({\mathrm{PO}}_{4}\right)}_{6-x}{\left(\mathrm{OH}\right)}_{2-x}\to {\mathrm{Ca}}_{10}{\left({\mathrm{PO}}_{4}\right)}_{6}{\left(\mathrm{OH}\right)}_{2}+{\beta \text{-Ca}}_{3}{\left({\mathrm{PO}}_{4}\right)}_{2}{+\mathrm{xH}}_{2}O$$

when the Ca/P molar ratio is 1.5 and x = 1 in Equation (1), the Ca_9_(HPO_4_)(PO_4_)_5_(OH) is named Ca-deficient hydroxyapatite (CDHA), which is chemically and compositionally similar to β-TCP [10]. The crystal structure of CDHA has not been completely identified yet, but the structural and chemical investigations indicate that CDHA exhibits a similar structure to the stoichiometric HA, with some calcium and hydroxide ions missing [26]. Water may substitute the vacant positions of hydroxide and calcium ions in the crystal lattice of CDHA [26]. Calcium deficiency is related to the preparation method, including the reaction parameters. Chemical transformation of CDHA to β-TCP via heat treatment can be described by Equation (2):

(2) $${\mathrm{Ca}}_{9}\left({\mathrm{HPO}}_{4}\right){\left({\mathrm{PO}}_{4}\right)}_{5}\left(\mathrm{OH}\right)\to {3\mathrm{Ca}}_{3}{\left({\mathrm{PO}}_{4}\right)}_{2}{+H}_{2}O$$

The phase transformation from CDHA to β-TCP was reported in the relevant literature [19,21,23,24,26,27]. Nevertheless, transforming the conventional CDHA phase, which is possible to obtain at lower temperatures, to β-TCP has often been reported to take place at high temperatures (>850 °C) accompanied by extended waiting times in excess of 8 to 10 h. However, almost no systematic discussion was reported for the formation of β-TCP considering both the reaction pH and sintering temperature. Considering the fact that low calcination temperatures are favored to obtain β-TCP with a finer morphology in adaptation to natural skeletal tissue, whether the pH can be an effective means to allow this has partly remained elusive. Motivated by this, we systematically explored the range of pH that we thought would allow low-temperature transformation of CDHA to β-TCP. The rationale behind this approach was that low pH precursor solutions sustain their stability for prolonged times and that the fine nanostructures expected to precipitate from such a solution, aided by the high surface energy they are expected to possess, could favor stabilization of the β-TCP phase at lower temperatures. We report a full transformation of CDHA to β-TCP at 750 °C in under 3 h from Ca^++^ and PO_4_^3−^ precursor solutions prepared under a pH of 5.5. The lower temperatures and the shorter sintering time allow for a fine nanostructured morphology with high crystallinity, likely eliminating the possibility of excessive grain growth that is otherwise expected to occur under high-temperature treatment with long process times. Low-temperature sintering of β-TCP could be desired for use as a biocompatible coating on an alloy-based load bearing application inside the body whereby the coating enhances the osteointegration to the host tissue. Such a lowering of the sintering temperature also aids in minimizing the high-temperature corrosion of the substrate alloy as most metals are prone to oxidation at elevated temperatures. In the pursuit of doing so, we investigated the formation of β-TCP nanoparticles from a simple, water-based sol-gel technique considering the synthesis pH and sintering temperature by using X-ray diffraction (XRD) and Raman spectroscopy. The morphology of the particles was also examined by transmission electron microscopy (TEM). We were able to show that low pH could aid in stabilizing the β-TCP phase at relatively low temperatures than those reported in previous works, rendering pH control of the initial precursor solution a prominent synthesis parameter. As such, the lowering of the sintering temperature to obtain β-TCP also helps in preserving the needle-like morphology of the as-dried product that is vital in enhancing its interaction with natural cells.

## 2. Materials and Methods

### 2.1. β-TCP Synthesis

β-TCP nanoparticles were synthesized by the water-based sol-gel technique, which was conducted according to a similar procedure, formerly reported by Bakan et al. [20]. All the reagents used in this study were analytical grade and supplied by Sigma-Aldrich. Briefly, Ca(NO_3_)_2_.4H_2_O and NH_4_H_2_PO_4_ were used as the Ca and P precursors, respectively, with a Ca/P molar ratio of 1.55. Both precursors were prepared using 18.2 MΩ cm deionized water. Prior to the reaction, the pH of both solutions was adjusted with ammonia (32%). The pH values of the synthesis reaction were chosen as 5.5, 6.5, 7.0, 7.5, and 8.0 for the investigation of the effect of pH on the formation and/or particle characteristics of β-TCP. While P precursor was stirred at 600 rpm at 45 °C, Ca precursor was added drop by drop using a peristaltic pump. After 24 h of aging at room temperature, the precipitated particles were collected by filtration and were then washed with deionized water to remove NH_4_^+^ and NO_3_^−^ ions. The filter cake was then dried overnight in a vacuum oven (Binder GmbH, Tuttlingen, Germany) at 80 °C.

**Figure 1:**
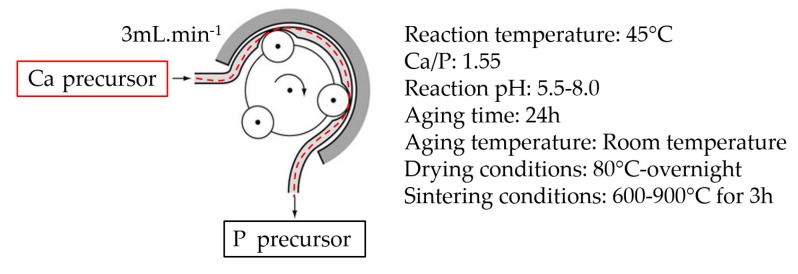
The scheme of the nanoparticle synthesis procedure.

Afterward, to complete the chemical conversion of CDHA to β-TCP, the dried powders prepared with the prescribed method above were placed in an Al_2_O_3_ crucible. The powders were subsequently heated to temperatures varying from 600 °C to 900 °C depending on the experiment and with a heating rate of 10 °C/min. The samples were held for 3 h at the desired temperature followed by rapid cooling down to room temperature. The scheme of the nanoparticle synthesis procedure is given in Figure 1.

### 2.2. Materials Characterization

To analyze the phase composition, X-ray diffraction (XRD) (Bruker, Billerica, Massachusetts, USA) analysis was carried out by a Bruker D2 Phaser using CuKα radiation at the step scanning mode, applying a tube voltage of 30 kV and a tube current of 10 mA. A step size of 0.02° and a scan speed of 1 s/step were chosen. Based on XRD peak broadening, the mean crystallite size (D) of the samples was calculated via the Scherrer’s equation using (002) and (0210) reflections corresponding to HA and β-TCP, respectively:

(3) $$D_{hkl}=0.94\lambda /\left(\mathrm{Cos}\theta \right)B_{hkl}$$

where B*_hkl_* is full width at half maximum in radians, λ is the wavelength of Cu Kα radiation (1.5406 Å), k is the shape factor that is equal to 0.94, and θ is the diffraction angle of the corresponding reflection plane [20,29,30]. The degree of crystallinity (*X*c) corresponds to the percentage of crystalline phase in the investigated volume of a sample. Similar to D, *X*c can be calculated from the width of a corresponding reflection:

(4) $$X_{C}={\left(K_{A}/B_{hkl}\right)}^{3}$$

where X_C_ is the degree of crystallinity, B*_hkl_* is the full width of the peak at half maximum in degree, and *K*A is a constant fixed at 0.24 [31].

In addition, Raman spectroscopy enabled differentiation of HA and β-TCP at the molecular level, even when the degree of crystallinity of the samples was poor. Raman spectroscopy (Renishaw, Wotton-under-Edge, UK) measurements were performed using a Renishaw Raman InVia System coupled with a 532 nm green laser to identify the characteristic (PO_4_)^3−^ groups that are present in the samples obtained using different parameters. Transmission electron microscopy (JEOL, Tokyo, Japan) (TEM, JEOL-JEM-2100F UHR7HRP), operating at an accelerating voltage of 200 kV, was used to examine the powder morphology. For TEM analysis, the samples were ultrasonically suspended in ethanol and then were dropped on the TEM grid, which was subsequently dried for the analysis.

### 2.3. Cytotoxicity Assay

The effect of synthesized β-TCP particles on the cell viability of human osteoblast-like cell line MG-63 (ATCC, Rockville, USA) was examined using MTT colorimetric assay. The cells were seeded into 96-well plates at a density of 5 × 10^4^ cells/mL. Cells were grown in Dulbecco’s modified Eagle’s medium (DMEM) supplemented with 10% fetal bovine serum (FBS) containing 1% L-glutamine 200 mM and 1% penicillin/streptomycin in a humidified 5% CO_2_ incubator at 37 °C. Prior to the introduction of the particles to the cells, the particles were irradiated for 30 min under the UV light. β-TCP nanoparticles (100 µg/mL) were prepared by making the dilutions with DMEM cell culture media. The samples were incubated for 24 h, 48 h, and 72 h. After the incubation, each well was replaced with 100 µL of fresh media and 13 µL of thiazolyl blue tetrazolium bromide (MTT) reagent (5 mg/mL, diluted with PBS). The plates were further incubated in the dark at 37 °C for 4 h. Then, the media were removed and 100 µL of dimethyl sulfoxide (DMSO) was added to the wells to dissolve the insoluble formazan crystals formed after 4 h. The concentration of formazan crystals produced in the viable cells was determined by measuring the absorbance at 570 nm using a microplate reader. The cells treated with only fresh media served as a control. The percentage of cell viability of each group was calculated by assuming the cell viability of the control as 100%.

The data are presented as the mean ± standard deviation (SD) of four repetitions. The significant differences between the groups were determined using one-way analysis of variance (ANOVA) and Student’s *t*-test. Statistical analysis was performed using IBM SPSS Statistics software version 23.0 (SPSS Inc., Chicago, IL, USA). All *p* values less than 0.05 were considered to be statistically significant.

Please see Table S1 in the Supplementary Materials for all analyses conducted on the prepared samples.

## 3. Results and Discussions

### 3.1. Characterization of β-TCP Nanoparticles

#### 3.1.1. XRD

**Figure 2:**
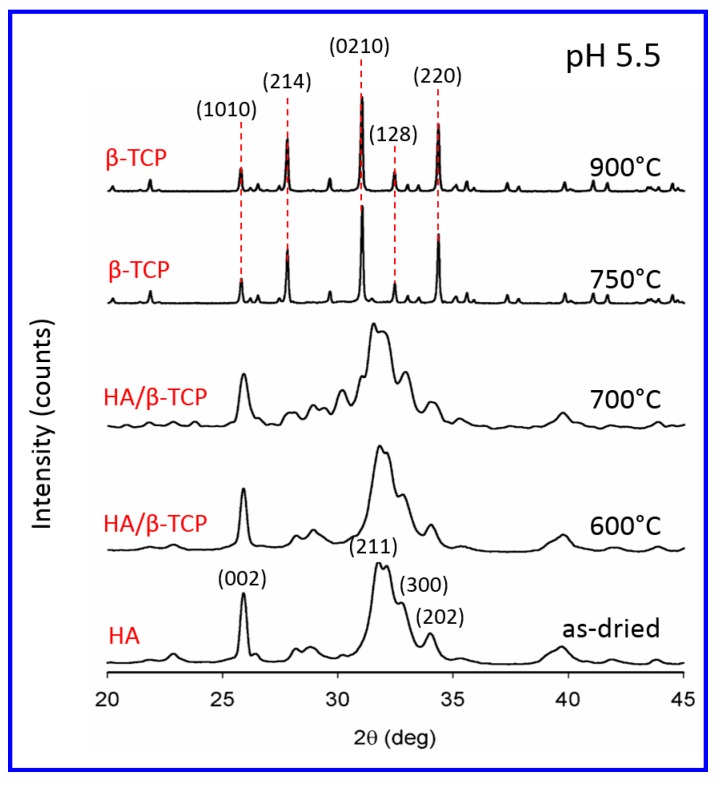
XRD patterns of the samples that were synthesized at pH 5.5. The effect of the sintering temperature on the chemical transformation of Ca-deficient hydroxyapatite (CDHA) to β-tricalcium phosphate (β-TCP) is revealed. Transformation to β-TCP is almost complete as early as 750 °C.

In Figure 2, the XRD patterns of the as-dried sample and the samples that were sintered at different temperatures are given for the pH 5.5 synthesis condition. XRD patterns were identified taking the Joint Committee on Powder Diffraction Standards (JCPDS) No:09-0432 for HA and No: 09-0169 for β-TCP into account. The XRD patterns revealed a transformation signaled by the disappearance of the broad peaks of CDHA and, simultaneously, the appearance of the sharp peaks of β-TCP as early as at 750 °C. The corresponding phase analyses confirmed that all major peaks of β-TCP were present in the spectra (Figure 2).

**Figure 3:**
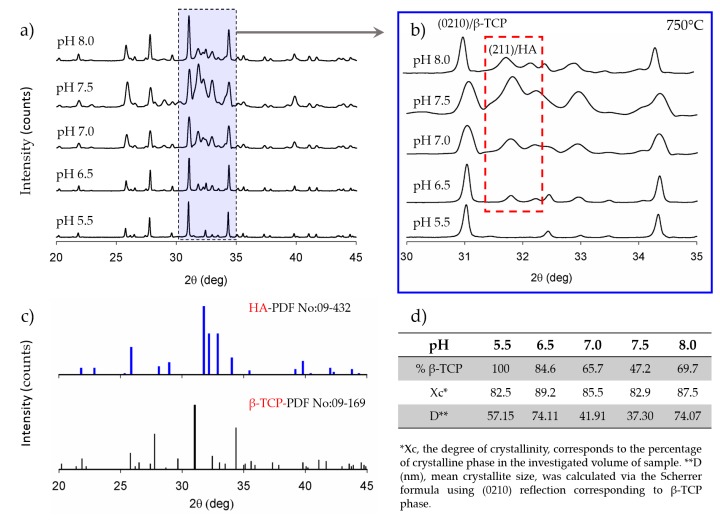
XRD results of the samples that were sintered at 750 °C: (**a**) For the range of 20–45°; (**b**) for the range of 30–35°; (**c**) XRD pattern of standard HA (No:09-0432) and β-TCP (No: 09-0169); (**d**) calculated percentage of transformed β-TCP, average crystallite size, and the degree of crystallinity. The effect of sintering temperature on the chemical transformation of CDHA to β-TCP is revealed. Transformation to β-TCP is completed as early as 750 °C for the sample that was synthesized under pH 5.5.

**Figure 4:**
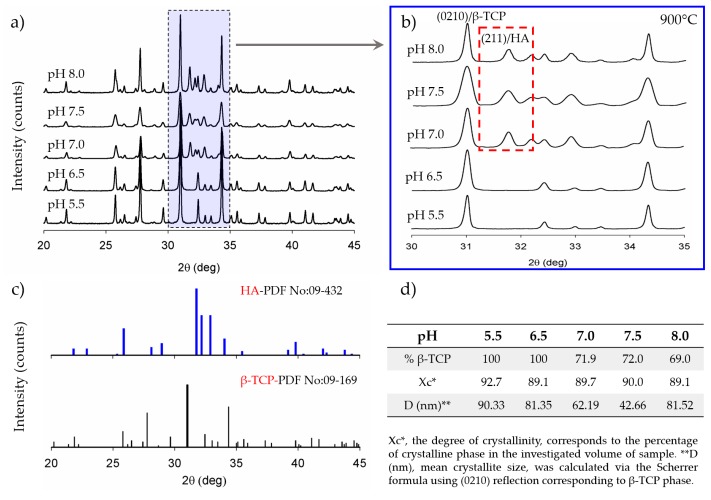
XRD investigation of the samples that were sintered at 900 °C: (**a**) For the range of 20–45°; (**b**) for the range of 30–35°; (**c**) XRD spectra of standard HA (No:09-0432) and β-TCP (No: 09-0169); (**d**) calculated percentage of transformed β-TCP, average crystallite size, and the degree of crystallinity. The transformation from CDHA to β-TCP was completed for the samples that were synthesized under pH 5.5 and pH 6.5.

The effect of pH on the formation of β-TCP is presented in Figure 3a,b and Figure 4a,b, for two different sintering temperature values. For each reaction pH and the related sintering condition, the percentage of transformed β-TCP, the average crystallite size, and the degree of crystallinity were quantified from the area using characteristic peaks via DIFFRAC.EVA software (Bruker AXS GmbH, Karlsruhe, Germany) and are given in Figure 3d and Figure 4d.

**Figure 5:**
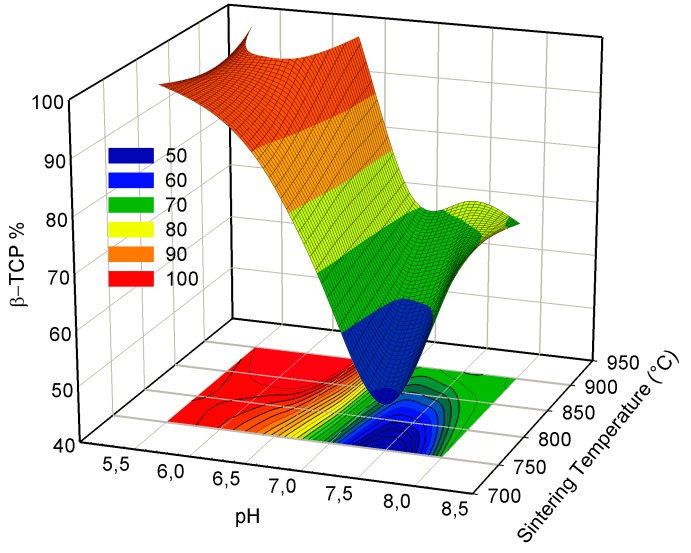
The percentage of transformation depending on the reaction pH and sintering temperature. Among all the synthesis and sintering conditions, a complete transformation of CDHA to β-TCP was only observed for the pH 5.5/750 °C and pH 5.5–6.5/900 °C samples.

For pH values higher than 6.5, CDHA can only partially transform into β-TCP and only at temperatures higher than 750 °C. Therefore, as can be seen from Figure 5, among all the synthesis and sintering conditions, a complete transformation of CDHA to β-TCP was only observed for the pH 5.5/750 °C and pH 5.5–6.5/900 °C samples. In the relevant literature, an almost complete transformation for higher pH values and at high temperatures was reported only after extensive dwell times much longer [19,23,26] than what was intended in our work.

It is likely that by decreasing the pH, and thereby depleting the concentration of OH^−^ ions, the stoichiometry required to form HA is altered in favor of β-TCP formation. This scenario was reported in the relevant literature [19,23,26] for adjusted Ca amounts, however, for fixed pH. While the OH^−^ depletion could explain the shift towards β-TCP from a stoichiometry point of view, the reduction in the sintering temperature remains elusive. Temperatures more than 900 °C–1000 °C are commonly reported to obtain a 100% β-TCP phase, however, we have not come across any prior work reporting the temperature ranges of β-TCP formation we report here (in the 720 °C–750 °C range) with process durations of only a few hours. The latter implies a change in the kinetic parameters induced by the OH^−^ depletion, which could emanate from the ease with which the nucleation of the β-TCP centers in a predominantly OH^−^ depleted CDHA start, eventually resulting in reduced process temperatures. Similar temperature ranges were considered for the heat treatment to obtain the pure β-TCP phase. However, only with a prolonged dwell time (as long as 30 h) was possible to acquire only a fractional transformation into β-TCP from the precursor solution [23].

#### 3.1.2. Raman Spectroscopy

**Figure 6:**
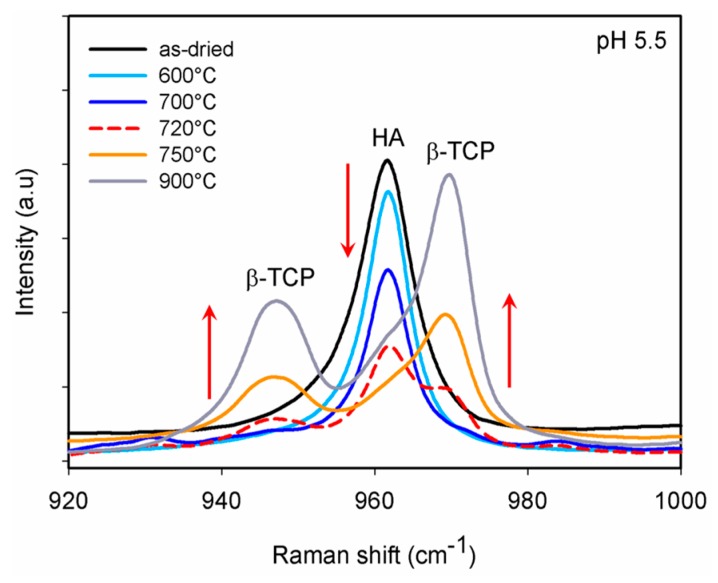
Raman spectra of the samples that were synthesized at pH 5.5. The effect of sintering temperature on the chemical transformation of CDHA to β-TCP is revealed by tracking the most characteristic ν1 mode. Transformation to β-TCP is started at 720 °C and is almost complete as early as 750 °C.

**Figure 7:**
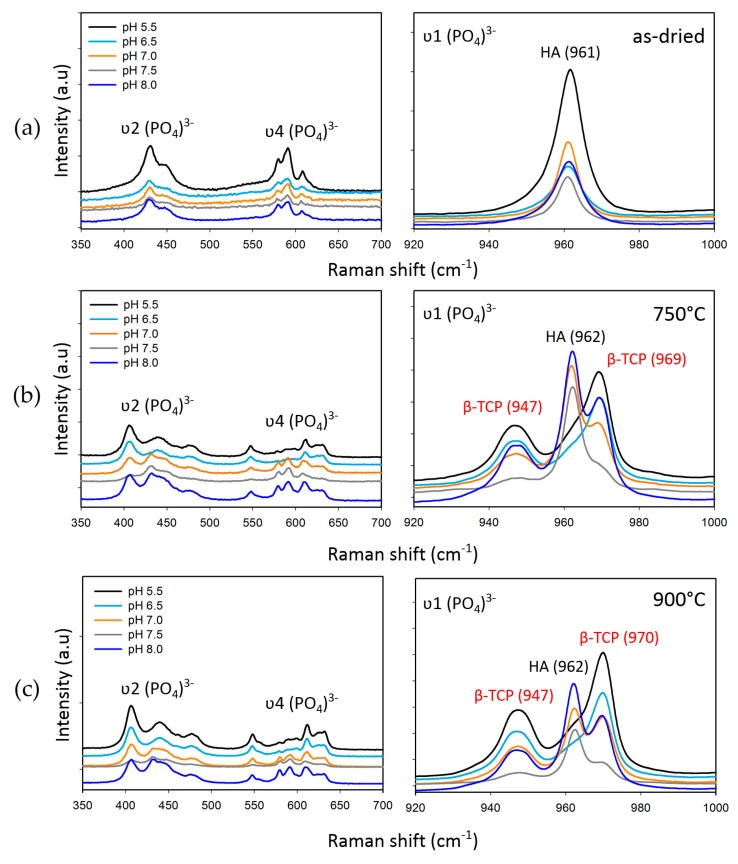
Raman spectra of the samples obtained at different conditions of pH and sintering temperature. The alterations in the (PO_4_)^3−^ ν1, ν2, and ν4 vibration bands for each synthesis condition are given: (**a**) Corresponding vibration bands of the as-dried samples; (**b**) corresponding vibration bands of the samples sintered at 750 °C; (**c**) corresponding vibration bands of the samples sintered at 900 °C.

Following the XRD analysis, we carried out Raman spectroscopy on our samples to identify the early stages of the transformation, and especially to confirm the formation of β-TCP at 720 °C. XRD patterns for the samples sintered at 720 °C posed a difficulty given the complexity of the unit cells of the two phases, rendering a direct assessment of β-TCP formation difficult. Raman, on the other hand, owing to the distinct vibrational modes of the two phases, can provide a better assessment of the early stages of the formation of the β-TCP phase from the CDHA. As HA and β-TCP are dominated by the (PO_4_)^3−^ group modes, therefore, the Raman spectra of the samples were evaluated, particularly taking internal (PO_4_)^3−^ modes into consideration. While β-TCP has 42 (PO_4_)^3−^ tetrahedra in the unit cell, the HA unit cell contains only six (PO_4_)^3−^ tetrahedra [28,32]. Due to the higher number of the phosphate groups in the unit cell of β-TCP, a greater number of vibrational modes is expected. The initiation of β-TCP formation of the samples that were synthesized under pH 5.5 is revealed in Figure 6. The characteristic band at 962 cm^−1^ is assigned to the totally symmetric stretching mode (υ1) of the tetrahedral (PO_4_)^3−^ group (P−O bond) in the case of HA, whereas in β-TCP, the same stretching mode can be observed at 969 cm^−1^ [28,32]. Besides, a wide band at 946–949 cm^−1^ appeared in the same spectrum, which belongs to the υ1 mode, but yielding a pattern with distinctly split peaks [28]. Therefore, the formation at 720 °C was detected only by analysis of the Raman spectra, which was not possible to deduce from the XRD patterns. The comparative Raman spectra of the samples that were prepared using different conditions of pH and sintering temperatures are presented in Figure 7. In addition to the υ1 mode, triple (υ4) and doubly (υ2) degenerate bending modes (O−P−O bond) were observed in the region of 570–625 cm^−1^ and 400–490 cm^−1^, respectively [28,32]. Unlike the well-separated *v*2 and *v*4 modes in the spectrum of HA, these modes appeared very close together in a broader range in the β-TCP spectrum. The difference between the number of phosphate groups in the unit cells of HA and β-TCP and therefore the variety of vibration bands leads to distinct Raman scattering from the two structures, allowing one to clearly identify the two phases. This is especially important when searching for the onset of the transformation of the β-TCP from CDHA, which was not possible to deduce from the XRD results as aforementioned.

#### 3.1.3. Morphology

**Figure 8:**
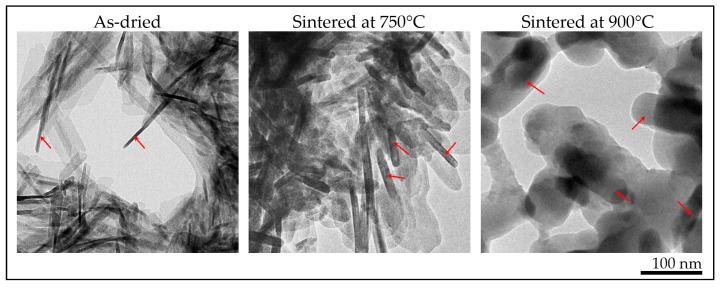
Bright-field TEM micrographs of the as-dried and sintered samples, which were synthesized at pH 5.5. After sintering at 750 °C, pure β-TCP is obtained, keeping its initial as-dried morphology with slightly thicker needle-like structures.

In Figure 8, bright-field TEM micrographs of β-TCP particles that were synthesized at pH 5.5 are given. As-dried CDHA particles are observed to be existing in needle-like morphology rather similar to that of stoichiometric or nonstoichiometric HA synthesized by wet-chemical processing [16,20,23]. After sintering at 750 °C, phase transformation was completed, and the obtained β-TCP particles became slightly thicker while maintaining their as-dried morphology. As the sintering temperature reaches 900 °C, particle coarsening occurs where the aspect ratios changed dramatically since the diffusive process dominates at such temperatures, maximizing the volume while minimizing the surface area (compare the 750 °C and 900 °C sintered structures in Figure 8).

### 3.2. Cell Viability

**Figure 9:**
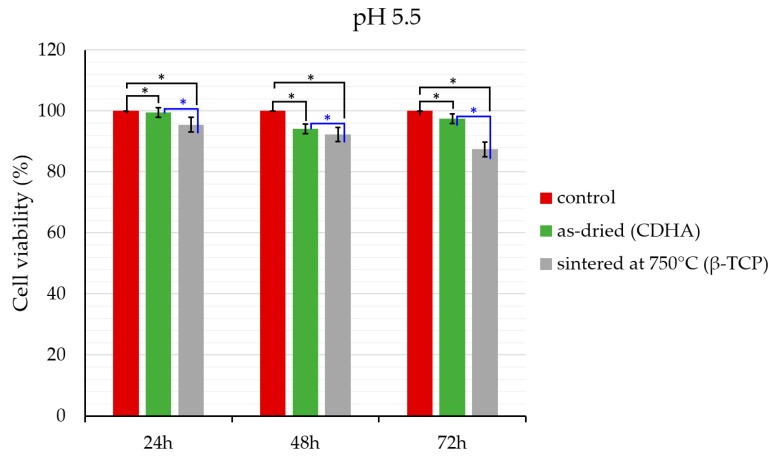
The effect of β-TCP particles (100 µg/mL) on cell viability of MG-63 osteoblast-like cells. The cells treated with only cell culture media (Dulbecco’s modified eagle medium) served as a control. The results represent the mean (±standard deviation) of four independent experiments. One-way ANOVA analysis was performed to confirm the statistical significance between the control and the other samples at the relevant time points while the significant difference between CDHA and β-TCP was statistically analyzed by Student’s *t*-test (*p* < 0.05).

Cell cytotoxicity evaluation of β-TCP particles that were synthesized at pH 5.5 is given in Figure 9. In all cases, the highest cell survival was observed at the lowest incubation time, and there was a time-dependent cell toxicity increase. However, the results demonstrated that before and after sintering, the particles had almost no cytotoxicity against MG-63 cells. Cell viability of the sintered samples was lower than that of the as-dried ones for all incubation times. As β-TCP is more soluble than CDHA, β-TCP dissolved more than CDHA in the cell culture media and this might cause a slight increase in the concentration of calcium ions. Calcium ions take part in a broad range of vital cell functions in eukaryotic cells. The increase in calcium concentration may damage the cell membrane by disturbing the state of cell electrolytes and it can also trigger programmed cell death [33,34,35], hence the slight decrease in cell viability in the samples that transformed fully to the β-TCP.

## 4. Conclusions

In this work, the effect of reaction pH and sintering temperature on the formation of β-TCP was examined systematically. Fully transformed β-TCP particles with a fine nanosized needle-like morphology were obtained from the chemical transformation of CDHA, which was synthesized by the wet precipitation method. As β-TCP is a high-temperature phase, it cannot be obtained directly from aqueous solutions and is stabilized usually only after extensive heat treatment. In the corresponding literature, the full transformation from CDHA to β-TCP was reported to occur relevantly at rather high sintering temperatures (>800 °C–900 °C) and only after prolonged dwell times. Our findings, however, show that the full transformation from CDHA to β-TCP is possible as early as 750 °C for the sample synthesized under a pH value of 5.5. Such an outcome indicates the importance of the pH of the precursor solution as one of the initial parameters, a point apparently overlooked in previous works, where the pH was often fixed at relatively high values. As checking the effect of pH on the transformation percentage of β-TCP was pursued in this work, experiments were also carried out with higher pH values than 5.5 to reveal low-temperature synthesis feasibility. For pH values higher than 6.5, CDHA can only partially transform into β-TCP and only at temperatures higher than 750 °C. The transformation from CDHA to β-TCP was tracked using both XRD and Raman spectroscopy as XRD results were inconclusive for detecting the onset of CDHA to β-TCP transformation at low sintering temperatures (at ≈720 °C). Raman spectroscopy provided a better assessment of the early stages of the formation of the β-TCP phase, owing to the distinct vibrational modes of CDHA and β-TCP. Bright-field TEM images revealed the presence of a fine needle-like β-TCP phase obtained at 750 °C, maintaining almost the initial as-dried morphology. High temperatures led to coarsened, low aspect ratio particles of β-TCP, resulting in the total loss of initial needle-like morphology, an outcome that could limit its effective incorporation into natural skeletal cells or applications that require cellular uptakes, such as drug or nucleic acid delivery. The procedure reported herein could pave the way to lower the transformation temperature of CDHA to β-TCP, which is often desired to produce fine nanostructures compatible with skeletal tissues in addition to avoiding excessive exposure of load bearing implant alloys to high temperatures during coating.

## Supplementary Materials

The following are available online at http://www.mdpi.com/1996-1944/12/3/354/s1, Table S1: Summary of the experimental conditions analyzed. The grey shaded cells imply the analysis conducted. Among all the synthesis and sintering conditions, a complete transformation of CDHA to β-TCP was only observed for the pH 5.5/750 °C and pH 5.5–6.5/900 °C samples shown with dashed red frames. Therefore, further investigations were only carried out for the samples that had been synthesized at pH 5.5.
