# Supplementary material for: A Systematic Study of the Effect of pH on the Initialization of Ca-deficient Hydroxyapatite to β-TCP Nanoparticles
Source: Materials (Basel). 2019 Jan 23;12(3):354. doi: 10.3390/ma12030354 (PMC6384749; doi:10.3390/ma12030354)
Supplement: Supplementary file 1 [file materials-12-00354-s001.pdf]

Table S1. Summary of the experimental conditions analyzed. The grey shaded cells imply the analysis conducted. Among all the synthesis and sintering conditions, a complete transformation of CDHA to β-TCP was only observed for the pH 5.5/750°C and pH 5.5-6.5/900°C samples shown with dashed red frames. Therefore, further investigations were only carried out for the samples that had been synthesized at pH 5.5.

	**Samples**																
	**pH 5.5**						**pH 6.5**			**pH 7.0**			**pH 7.5**			**pH 8.0**	
**Analysis Technique**	AD	S600	S700	S720	**S750**	S900	AD	S750	S900	AD	S750	S900	AD	S750	S900	AD	S750	S900
XRD					β-TCP	β-TCP			β-TCP									
Raman Spec.					β-TCP	β-TCP			β-TCP									
TEM																		
MTT																		

AD: as-dried **S600**: sintered at 600°C **S700**: sintered at 700°C **S720**: sintered at 720°C **S750**: sintered at 750°C **S900**: sintered at 900°C
